# Supplementary material for: Complexome profiling of the Chlamydomonas psb28 mutant reveals TEF5 as an early PSII assembly factor
Source: Plant Cell. 2025 Mar 17;37(6):koaf055. doi: 10.1093/plcell/koaf055 (PMC12164590; doi:10.1093/plcell/koaf055)
Supplement: koaf055_Supplementary_Data [file koaf055_supplementary_data.zip › psb28_interactive.zip!/plots/intensity/qualityPlots/peptides/_Cre12.g556250.t1.2.html]

### Cre12.g556250.t1.2

Trivial name:   
SeqCoverage [median]: 33.50  
Mass: 54.24 kDa  
  
 

| Sequence | MissedCleavage | Mass [Da] | wtIntensity | muIntensity | Score |
| --- | --- | --- | --- | --- | --- |
| ADTMTIR | 0 | 806.40 | 2068.98 | 3739.33 | 132.09 |
| AGVRMEDVR | 1 | 1031.52 | 325.84 | 790.66 | 120.12 |
| ALLMKEALEEISK | 1 | 1473.81 | 0.00 | 4557.75 | 92.04 |
| ASEMVFGK | 0 | 867.42 | 586.49 | 1185.47 | 111.82 |
| EALEEISK | 0 | 917.47 | 3341.32 | 976.71 | 83.75 |
| EANTYR | 0 | 752.35 | 733.96 | 748.64 | 107.55 |
| EANTYRTEVANR | 1 | 1422.69 | 443.13 | 4343.37 | 95.40 |
| FIGNNVDR | 0 | 933.47 | 1876.96 | 8743.00 | 104.45 |
| IANPMVPGIHDK | 0 | 1290.68 | 1486.98 | 1005.62 | 77.59 |
| IQDTPGYGDELDVFR | 0 | 1723.81 | 3474.83 | 0.00 | 97.91 |
| LIVVGDSGLGK | 0 | 1056.62 | 5429.14 | 20916.26 | 110.87 |
| LQVHDGSYTPTEQFR | 0 | 1776.84 | 3969.17 | 7041.01 | 72.90 |
| LQVHDGSYTPTEQFRR | 1 | 1932.94 | 0.00 | 1063.98 | 54.60 |
| MVQDYIESQNRK | 1 | 1509.72 | 2106.03 | 3387.74 | 68.36 |
| SLISIPGER | 0 | 970.54 | 7592.59 | 15315.26 | 107.01 |
| WLELEQAR | 0 | 1043.54 | 0.00 | 1083.90 | 65.04 |
| YLNNHSGPNDFAMAAPATEEK | 0 | 2276.02 | 0.00 | 926.29 | 46.36 |
|  |  |  | 33435.4 | 75825.0 |  |
